# Wonder symphony: epigenetics and the enchantment of the arts

**DOI:** 10.1093/eep/dvae001

**Published:** 2024-02-01

**Authors:** Marta Gallazzi, Marta Pizzolante, Elia Mario Biganzoli, Valentina Bollati

**Affiliations:** Catholic University of Milan, Milan 20123, Italy; EPIGET LAB, Department of Clinical Sciences and Community Health, University of Milan, via San Barnaba 8, Milan 20122, Italy; Research Center in Communication Psychology (PSICOM), Department of Psychology, Catholic University of Milan, Milan 20123, Italy; Unit of Medical Statistics, Bioinformatics and Epidemiology, Department of Biomedical and Clinical Sciences (DIBIC), University of Milan, Via Giovanni Battista Grassi, 74, Milan 20157, Italy; Unit of Clinical Research and Medical Statistics, Ospedale “L. Sacco” LITA Campus, Via Giovanni Battista Grassi, 74 Milan 20157, Italy; EPIGET LAB, Department of Clinical Sciences and Community Health, University of Milan, via San Barnaba 8, Milan 20122, Italy; Occupational Health Unit, Fondazione IRCCS Ca’ Granda Ospedale Maggiore Policlinico, Via San Barnaba 8, Milan 20122, Italy

**Keywords:** arts, music, positive experiences, epigenetics, well-being, health

## Abstract

Epigenetics, the study of heritable changes in gene expression without altering the DNA sequence, has gained significant attention due to its implications for gene regulation and chromatin stability. Epigenetic mechanisms play a fundamental role in gene–environment interactions, shaping individual development and adaptation. DNA methylation, histone modifications, and non-coding RNAs are key epigenetic regulators. Epigenetic changes can be triggered by environmental factors, including stress, toxins, and social interactions, influencing health and well-being. Positive experiences, such as engagement with the arts, have been linked to emotional responses and neurotransmitter release. While the impacts of detrimental factors on epigenetics have been widely studied, the effects of positive influences are less explored. Specifically, visual art and music have profound effects on emotions, cognition, and mood regulation. Exposure to arts enhances memory, reduces stress, and fosters social inclusion. Recent research has begun to explore the links between positive experiences and epigenetic modifications, suggesting that aesthetic experiences, including visual art and music fruition, might induce dynamic and/or stable changes in gene expression profiles. However, this field is in its infancy, and more research is needed to establish clear connections. Collaborative efforts among genetics, epigenetics, neuroscience, psychology, and the arts are essential for a comprehensive understanding. Longitudinal studies tracking sustained exposure to positive experiences and examining the influence of childhood artistic education on the biological bases of therapeutic effects of art and music are promising avenues for future research. Ultimately, understanding how positive experiences influence epigenetics could provide insights into the long-term enhancement of human well-being.

## Introduction

The epigenome represents the plasticity layer related to gene regulation essential to determine the dynamic adaptive mechanisms of life in the environment. Epigenetics is the study of heritable changes in gene expression that occur without altering the underlying DNA sequence [1]. This growing field of study has obtained substantial attention in recent decades for its profound implications for understanding the regulation of gene expression and chromatin stability [2]. Moreover, epigenetic mechanisms orchestrate the interplay between genes and the environment, allowing ability of the cells to respond to external stimuli and shaping individual development and adaptation to their surroundings [3, 4].

The two primary mechanisms of epigenetic regulation are DNA methylation and histone modifications, which interact to shape the chromatin landscape. Often, non-coding RNAs (ncRNAs) are reported as epigenetic mechanisms, even though they slightly deviate from the traditional definition of epigenetics.

DNA methylation involves the enzymatic addition of methyl groups to cytosines located in CpG dinucleotides. This process silences gene expression by hampering access to the transcriptional [5]. Histone modifications include acetylation, methylation, phosphorylation, and ubiquitination, and together, they define structural and functional changes in chromatin organization. Among the various modifications, histone acetylation stands as one of the most extensively investigated. It plays a critical role in promoting an open chromatin configuration, thereby facilitating gene activation and fostering robust transcription [6]. ncRNAs are RNA molecules that do not encode proteins but play crucial roles in regulating various cellular processes, ranging from gene expression regulation to chromatin remodeling [7]. There are several types of ncRNAs, including microRNAs (miRNAs) and long non-coding RNAs. miRNAs, for example, function as post-transcriptional regulators by binding to target mRNA sequences, leading to mRNA degradation or translational repression [8]. Long non-coding RNAs, on the other hand, can modulate gene expression through various mechanisms, such as guiding chromatin-modifying complexes to specific genomic loci [9].

Intriguingly, the inheritance of epigenetic information across generations has also garnered significant attention. Evidence suggests that certain epigenetic marks can be transmitted from parents to offspring, potentially influencing phenotypic traits and disease susceptibilities in subsequent generations [10].

Epigenetic changes triggered by detrimental environmental factors, such as environmental pollutants [3], endocrine-disrupting chemicals [11], stress [12], imbalanced diet [13], tobacco [14], alcohol [15], viral and bacterial infections [16], sedentary lifestyles [17], childhood trauma [18], and social isolation [19], have been widely investigated. However, epigenetic marks are highly plastic, and they serve not only as a mechanism of damage but also as a means of supporting cellular adaptation [20]. Remarkably, the epigenetic landscape is predominantly shaped by all life experiences, with a profound influence observed for those of a positive nature. Unfortunately, compared to detrimental factors, epigenetic modifications induced by positive experiences have received significantly less attention in scientific research. As a result, there is still much to explore and understand about the potential beneficial effects of positive influences on epigenetic regulation. Nonetheless, the emerging evidence highlights the importance of investigating both the damaging and advantageous aspects of epigenetic plasticity to comprehensively understand its impact on human health and well-being.

The purpose of this review is to present the current evidence on the intriguing relationship between art, music, beauty, well-being, and epigenetics, exploring their common background and mutual influence.

## Aesthetic encounter and the common background of positive experiences

Aesthetic experiences play a pivotal role in human life, encompassing encounters and interactions with a wide array of artistic forms. Such experiences can be elicited through various media, ranging from traditional forms like paintings, sculptures, music, plays, and films to more innovative ones, including immersive technologies [21]. Despite the diverse contexts and modalities through which these experiences occur, they all share a common background in evoking specific emotions [22], stimulating the senses [23], and offering a means of creative actions [24].

Among these diverse artistic forms, visual art (i.e. paintings and sculptures) and music, as distinct forms of creative expression, have been extensively investigated, making them highly relevant to the objectives of this study. Their prominence in the realm of multidisciplinary research, at the crosslink among the fields of psychology, neuroscience, and the arts, stems not only from their historical and cultural significance but also from the depth of psychological and neuroscientific insights they provide [24].

For instance, both visual art and music fruition have been shown to engage brain regions involved in emotion processing, reward, and aesthetic appreciation [25, 26]. Neuroimaging studies have provided evidence of heightened activity in the brain’s mesolimbic system, including the ventral striatum and the orbitofrontal cortex, during the fruition of both visual art and music [26, 27].

The aesthetic appreciation of visual art and music is inherently subjective, shaped by individual differences, cultural backgrounds, and personal preferences, rendering it a uniquely fascinating facet of human perception [28]. Correspondingly, when exposed to the same artwork or musical piece, different individuals may experience a diverse array of emotional responses. These reactions can range from positive emotions like joy, wonder, and happiness to negative ones such as sadness or even feelings of hate and anger. All these emotional responses, whether positive or negative, contribute collectively to the individual’s perception of beauty [29, 30].

The impact of visual art and music on mood regulation and stress reduction has also been well documented. Controlled studies have indicated that visual art and music fruition can induce relaxation and decrease stress and anxiety levels [31]. These effects are thought to be mediated by the release of neurochemicals, such as dopamine and endorphins, which are associated with feelings of pleasure and relaxation [32].

Furthermore, within the healthcare domain, art and music therapy (MT) have demonstrated clinical efficacy in supporting emotional expression and promoting healing. Therapeutic interventions involving art have been utilized to address a variety of psychological conditions, including depression, anxiety disorders, and post-traumatic stress disorder [33]. These therapies provide individuals with a non-verbal means of communication and emotional processing, leading to improved psychological well-being. Additionally, research suggests that engaging with art and music can enhance cognitive abilities, such as attention, memory, and creativity [34]. Additionally, visual art and music have been found to improve cognitive performance and problem-solving skills, highlighting their potential as cognitive enhancers [35].

In the subsequent sections, we will delve into and examine the relationship between the appreciation of both music and visual arts and their impact on well-being. This exploration will specifically focus on elucidating the role that positive emotions play in this context.

## Music listening and well-being

The relationship between music and well-being has garnered increasing attention in scientific research. Music has demonstrated a profound impact on emotions, subjective experiences, and psychological functioning. On the other hand, the concept of well-being represents an optimal state of mental, emotional, and physical health, becoming a pivotal goal for individuals and healthcare professionals alike.

The therapeutic potential of music has been explored across various medical contexts, although a comprehensive systematic review of these studies is beyond the scope of this current review. Nonetheless, a few illustrative examples can provide insight into the range of applications for music-based interventions in healthcare.

An interesting review explored the potential of music as a non-pharmacological intervention for mechanically ventilated patients in the intensive care unit, considering studies from 2010 to 2022. Music significantly affected vital signs such as heart rate, blood pressure, and breathing, reducing pain intensity and anxiety levels. Additionally, music intervention enhanced sleep quality, decreased delirium occurrences, and improved cognitive function [36].

The impact of listening to nature-based music on anxiety, physiological measures, and adjustment to the prone position in conscious and hypoxemic coronavirus disease 19 (COVID-19) patients was also observed. Using a single-blinded randomized-controlled trial design, 64 patients were divided into an intervention group and a control group. The intervention group received nature-based music along with routine care while in the prone position, whereas the control group received routine care alone in the same position. The results indicated that the intervention group experienced a statistically significant reduction in state anxiety compared to the control group, together with an improvement in heart rate, oxygen saturation, and prone position time [37].

As hospitalization often triggers agitation in dementia patients due to the abrupt change in environment, a personalized music intervention was used to mitigate agitation in Alzheimer’s and related dementia patients. The potential of individualized music programs to effectively reduce agitation in dementia patients was supported by a significant decrease in agitation scores post-intervention [38]. Music interventions have also demonstrated potential to improve hospitalized patients’ sleep. A meta-analysis conducted on 10 studies involving 726 patients, with diverse music interventions varying in music selection, duration, and timing, showed that music significantly improved sleep quality compared to standard treatment [39].

Music has been reported as an effective non-pharmacological modulator of stress. A randomized controlled trial was recently conducted to investigate the impact of music on 106 pregnant women who had experienced prenatal loss (53 exposed to music and 53 controls). A significant reduction in subjective anxiety and salivary cortisol levels was observed for the music group, while post-traumatic growth and well-being scores improved significantly [40]. Moreover, a recent study explored the impact of self-selected music on the quality of life of family caregivers for cancer patients in palliative home care, using a randomized trial where the intervention group received personalized daily music sessions, while the control group received therapeutic training education. The results showed that the music intervention significantly improved caregivers’ quality of life and satisfaction with care [41].

The potential therapeutic benefits of music during cancer treatment for enhancing patients’ psychological and physical well-being were evaluated on 750 adult patients undergoing outpatient chemotherapy infusion. Participants were randomized into music (listening up to 60 min) or control groups, and patients listening to self-selected music experienced significantly improved positive mood while decreased negative mood and distress (excluding pain) after the intervention [42].

The use of preferred music as a pain relief intervention for older adults with low back pain has also been proposed. Twenty participants listened to their preferred music via a mobile app for 4 days showing a decrease in their average pain scores [43].

The beneficial impact of music listening extends to healthy individuals as well. An interesting study investigated how different emotional music influences motor sequence learning. Participants engaged in a task while listening to fearful, pleasant, or neutral music. Music-induced pleasure enhanced movement speed and efficiency but prolonged the movement time. Fear-conditioned music improved verbal recall of sequence order. According to the authors, emotional music impacts motor learning by enhancing different aspects: pleasure enhances motor performance, possibly through increased dopamine availability, while fear-conditioned music improves declarative knowledge through attentional circuit recruitment [44]. In 16 healthy volunteers, active and passive MT has been found to influence the autonomic nervous system (ANS) and the hypothalamic–pituitary–adrenal axis activity. Using heart rate variability measures, active MT decreased the sympathetic autonomic nervous system activity, while passive MT increased it [37].

To test the hypothesis that music listening primarily influenced the autonomic nervous system’s response to stress, 60 healthy female volunteers were exposed to a psychosocial stress test after being assigned to three conditions: relaxing music, the sound of rippling water, or rest without acoustic stimulation. A significant modulation in cortisol response was observed, with the highest levels in the relaxing music condition. Moreover, salivary alpha-amylase recovery was faster in the relaxing music group [45].

### Music, positive emotions, and physical activity

Sedentary behavior is associated with deleterious physical and psychological health outcomes. While regular physical activity can enhance various quality-of-life measures, such as social and emotional well-being and overall engagement with life, music can be used as a tool to increase involvement in physical activities. In fact, it is able to amplify the beneficial effect of physical activity as it can elicit positive emotions and distract athletes from unpleasant feelings associated with physical exertion and fatigue [46]. A systematic review summarizes the studies interested in understanding the effects of music listening on health-related exercise and physical activity. The 23 theoretical texts analyzed allow to state that music could promote behavioral change with increased exercise adherence and participation [47].

Different studies demonstrate the effectiveness of using music during physical exercise. For instance, 27 physical active subjects completed the Profile of Mood States and the State Anxiety Inventory before and after treadmill running in music and no music condition. Participants exercised at 75% of their heart rate reserve until voluntary exhaustion. The results indicated significant mean changes on tension, depression, fatigue, confusion, and state anxiety. Female subjects reported greater mean fatigue after exercising in the presence of music than in the no music condition [48].

The use of music listening across a range of physical activities can promote more positive affective valence, enhance physical performance, reduce perceived exertion, and improve physiological efficiency. In fact, music is associated with significant beneficial effects on affective valence, physical performance, perceived exertion, and oxygen consumption [49].

### Music and positive emotions

Positive psychology has been interested in understanding what factors enable people to maintain or increase well-being. Seligman used the positive emotion, engagement, relationships, meaning, and accomplishment (PERMA-V) model to identify areas that a person can hone in order to achieve high levels of well-being. The flourishing aspects are positive emotion, engagement, relationships, meaning, accomplishment, and vitality [50]. Flourishing is a combination of feeling good and functioning effectively; it is related to an optimal operating condition, and it is the opposite end of the spectrum of common mental disorders. An interesting review investigated the relationship between psychological well-being and music in order to claim that music practice and participation can positively contribute to one living a flourishing life by positively influencing all five of the PERMA factors [51].

Music soliciting different forms of entrainment is able to enhance the functionality of various endogenous, emotion-granting regulative processes, and drawing novel experiences out of us with an expanded complexity and phenomenal character [52]. This is possible because music can arouse emotions through various mechanisms, for example brain-stem reflexes, rhythmic entrainment, episodic memory, musical expectancy, and aesthetic judgment [53]. Music is often cited as an effective tool for regulating emotions and may also promote well-being because music facilitates one’s ability to regulate the experience and expression of emotions. An interesting study explored in 565 working adults the pathways by which music use for cognitive and emotional regulation purposes may predict mental health outcomes. The results showed that listening to music for cognitive and emotional regulation predicts flourishing in life via the mediation pathway of cognitive reappraisal even after controlling for trait affects [54].

Collectively, these studies underscore music’s potential as a therapeutic agent, enriching our understanding of its diverse applications and reaffirming its capacity to contribute significantly to the enhancement of well-being across populations. To date, however, the underlying molecular mechanisms that account for these positive effects remain elusive.

## Visual art and well-being

The appreciation of visual arts, deeply rooted in the realms of aesthetics and beauty, has increasingly been recognized as a powerful means to enhance well-being [31]. The engagement with visual arts encompasses a wide spectrum of stimuli and experiences, transcending passive enjoyment to include active contemplation and introspective reflection. Pioneering research in this field, including the comprehensive reviews by Mastandrea *et al*. [23], has not only underscored the emotional and rewarding aspects of aesthetic encounters but also delved into their neurological underpinnings.

Engagement with visual arts, such as paintings, sculptures, and photography, often elicits profound emotional responses ranging from awe and inspiration to nostalgia and deep self-reflection [55]. These emotional experiences are instrumental in cultivating empathy and strengthening social ties, enabling individuals to explore and understand their emotions more deeply. This emotional resonance is partly due to the release of neurotransmitters like dopamine [56] and oxytocin [57], which are linked to pleasure [58] and social bonding [59], respectively. Additionally, interaction with visual arts can bolster psychological resilience, acting as a safeguard against stress and anxiety, and fostering feelings of accomplishment and contentment, crucial for overall well-being [32, 33].

The World Health Organization has acknowledged the significant role of art-based approaches in enhancing health and well-being, recognizing their potential in both preventive and therapeutic contexts [60]. This perspective is supported by various studies highlighting the therapeutic effects of art museum visits, which have been shown to improve memory, alleviate stress, and encourage social inclusion [61]. The benefits of visual arts have been observed across diverse groups, including the elderly [62, 63], individuals with mental health conditions [64], dementia patients [65], and those experiencing social isolation [66]. Art settings are particularly effective in promoting well-being by facilitating positive reminiscence, self-exploration, and group interactions. Additionally, psychophysiological studies have found that visits to art museums can reduce stress levels, with exposure to figurative art notably decreasing systolic blood pressure, possibly due to its straightforward nature and reduced ambiguity [67].

These collective insights underscore the profound impact of visual arts as a multifaceted catalyst for not only mental but also physical well-being. Visual arts, through their capacity to evoke emotions, stimulate cognitive functions, and foster social connections, extend beyond mere aesthetic appreciation to emerge as a powerful tool for holistic healing and personal enrichment.

### Visual art and positive emotions

As for music fruition, within the positive psychology framework, the role of visual arts has gained significant attention for its ability to elicit positive emotions and its contribution to the individual’s overall well-being and happiness [50, 51].

The realm of visual arts, encompassing diverse forms such as painting, sculpture, and photography, plays a pivotal role in the elicitation of positive emotions, a key aspect of psychological well-being. Scientific literature consistently highlights the unique capacity of visual arts to invoke feelings of joy, wonder, and serenity. Studies by Silvia *et al*. [68] and Dalebroux *et al*. [69] provide empirical evidence supporting the positive emotional impact of engaging with visual artworks. These studies demonstrate that exposure to visual arts can activate the brain’s reward pathways, particularly the ventral striatum and the prefrontal cortex, regions known to be associated with the experience of positive emotions.

Furthermore, visual arts offer a unique sensory experience that can lead to an enhanced state of mindfulness, fostering a sense of presence and immersion in the moment [70]. This aspect of visual art engagement has been linked to the reduction of stress and anxiety, contributing to an overall state of emotional well-being [71]. For example, the contemplation of aesthetically pleasing artwork has been shown to induce a state akin to meditative absorption, where the viewer’s attention is fully captured, leading to a decrease in cognitive rumination and an increase in positive mood states [72].

In addition, the visual arts serve as a medium for positive social interactions and community engagement. Art exhibitions and community art projects provide opportunities for social connection and shared experiences, which are fundamental for emotional well-being [73]. These social dimensions of art engagement are particularly significant, as they facilitate not only individual emotional uplift but also collective joy and a sense of belonging [74].

In summary, the visual arts hold a profound potential for the promotion of positive emotions. Through their ability to engage the brain’s reward system, provide immersive and mindful experiences, and foster social connectivity, visual arts stand out as a significant contributor to emotional health and psychological resilience.

## Epigenetics and positive emotions

Exploring the intricate interplay between our genetics, epigenetic modifications, and emotional experiences shows fascinating insights into the complexity of human well-being. A few studies to date have explored the possible effect of these positive emotions on the epigenetic landscape.

The role of positive emotions in modulating epigenetics should be framed in the context of biological embedding of experience with the expected impact on adaptive mechanisms of species in the environment [75].

Literature on biological embedding and epigenetics is fairly covering negative experiences related to socioeconomic disadvantage, stress, and trauma. However, biological embedding and epigenetics of positive experiences were not considered so far in literature. Although this lack could be justified by a major interest in investigating risk factors for accelerated aging and chronic diseases for preventive purposes, there is a substantial need of investigation of the compensating effects of beneficial factors on individual and social homeostasis in an evolutionary perspective. Whereas the identification of causal links of negative experiences as risk factors is challenging because they cannot ethically be experimentally assigned in humans, the study of positive experiences as beneficial factors could be assessed through randomized experimental trials in full ethical framework.

The pioneering epigenome-wide association study conducted by Baselmans *et al*. [76] was conducted on subjects that were part of the longitudinal survey studies of the Netherlands Twin Register and participated in the Netherlands Twin Register biobank project between 2002 and 2011. Well-being was assessed by a short inventory that measures satisfaction with life. Notably, the genomic coordinates of these sites—cg10845147, cg01940273, cg03329539, cg09716613, cg04387347, and cg02290168—mapped in proximity to genes like NEURL1B, ALPPL2, N4BP2L1, ZFPM1, and ZNF687. The subsequent gene ontology analysis revealed a distinct enrichment of central nervous system categories within the subset of high-ranking methylation sites.

An equally captivating hypothesis has emerged, postulating that repetitive element methylation might serve as a mediator of positive emotions [77]. According to this hypothesis, genomic and epigenetic mechanisms governing repetitive elements could potentially shape the emotional (limbic) brain, ultimately influencing the intricate dynamics of our social interactions.

Further exploring the connections between epigenetics and emotions, a study focused on the epigenetic modification of the oxytocin receptor gene in human infancy [78]. The study investigated how variations in oxytocin receptor gene methylation levels can be tied to emotional face processing. Higher levels of oxytocin receptor gene methylation were correlated with heightened responses to negative facial expressions—fearful and angry—and conversely, reduced responses to positive facial expressions like happiness.

Expanding the horizons of this exploration, the Normative Aging Study conducted in the Greater Boston MA area explored into the potential associations between psychological factors and DNA methylation [79]. This prospective cohort study engaged 538–669 elderly men, assessing psychological features and DNA methylation measurements across multiple visits from 1999 to 2006. Psychological distress indicators such as anxiety, depression, and hostility were linked to positive correlations with DNA methylation levels in genes like intercellular adhesion molecule-1 and coagulation factor III, both integral to immune and inflammatory processes. Moreover, intriguing hints of associations emerged between psychological factors and DNA methylation in promoters of other genes like toll-like receptor 2 and inducible nitric oxide synthase, with lower consistency for glucocorticoid receptor (nuclear receptor subfamily 3, group C, member 1, NR3C1), interferon-γ, and interleukin 6 promoters.

## Epigenetics and aesthetic experiences: a focus on music and visual art

It is essential to recognize that the field of epigenetics and aesthetic experiences is still in its infancy, and more research is needed to establish clear and robust connections between specific epigenetic changes and aesthetic phenomena. However, a few studies explored the biological plausibility of an epigenetic response to aesthetic experiences.

In a very interesting review, Brigati *et al*. introduce the concept of social epigenetics, helpful to comprehend the profound impact of musical experiences [80]. As music is intimately linked with human emotions and health, some biochemical changes that might occur in response to music listening, highlighting the capacity of aesthetic experiences to echo through gene modulation. The concept of a “transmissible, biochemical change” encapsulates the idea that the effects of music extend beyond the transient moments of auditory pleasure. Instead, these experiences could potentially leave an imprint on our epigenetics, initiating a cascade of molecular events that shape our responses to emotions and interpersonal connections.

The impact of short-term music exposure was examined through a pilot study [81]. During the study, individuals were invited to engage in an acute listening experience (*n* = 37) or not (*n* = 7), specifically Wolfgang Amadeus Mozart’s Violin Concerto No. 3 in G major, K.216, with a duration of ∼20 min. Blood samples were collected from each participant prior to the commencement of the musical session and immediately after its conclusion. By analyzing blood miRNAs of these individuals, the music-exposed subjects exhibited the upregulation of six miRNAs (hsa-miR-132-3p, hsa-miR-361-5p, hsa-miR-421, hsa-miR-23a-3p, hsa-miR-23b-3p, and hsa-miR-25-3p) and downregulation of two miRNAs (hsa-miR-378a-3p and hsa-miR-16-2-3p). Several of the upregulated miRNAs were associated with neuronal activity (miR-132, miR-23a, and miR-23b) and modulators of neuronal plasticity, cognitive functions, and memory, suggesting potential impacts on neurological health and function. The study also highlighted the roles of miR-132 and endoribonuclease Dicer upregulated after music listening, in protecting dopaminergic neurons and maintaining dopamine levels.

The same research group also investigated the impact of a 2-h classical music performance on the miRNA expressions in the peripheral blood of professional musicians, contrasting it with a control activity of equal duration that did not involve music [82]. After music performance, five miRNAs showed an increase in expression—hsa-miR-3909, hsa-miR-30d-5p, hsa-miR-92a-3p, hsa-miR-222-3p, and hsa-miR-30a-5p—while two miRNAs displayed a decrease—hsa-miR-6803-3p and hsa-miR-1249-3p. Interestingly, hsa-miR-222-3p and hsa-miR-92a-3p emerged as potential regulators of forkhead box P2 is a gene implicated in the development of linguistic abilities. Notably, miR-222 and miR-92 activation foster neurite outgrowth by dampening the neuronal growth inhibitor Phosphatase and tensin homolog and promoting cAMP-response element binding protein expression and phosphorylation, with an important role in memory formation, motor neuron functions, and neuronal plasticity. The elevation of miRNAs that are recognized regulators of auditory and nervous system functions—such as miR-30d, miR-92a, and miR-222—paints a vivid picture of the sensory and perceptual intricacies entwined with music performance [83].

**Figure 1:**
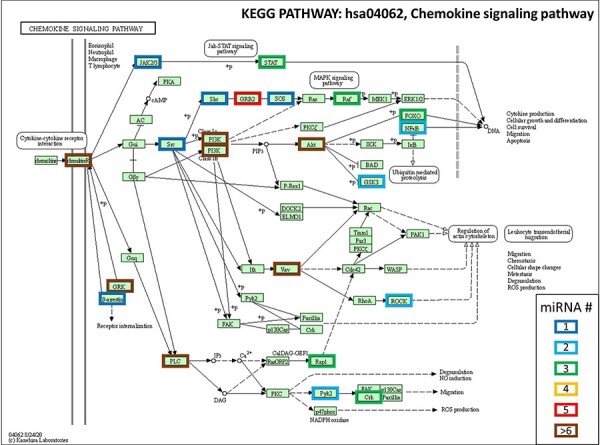
chemokine signaling pathway (hsa04062) as outlined in the KEGG database. For each gene reported in the pathway, the possible regulatory role of each miRNA highlighted in [66] and [67] is reported. The color of the gene frames is indicative of the number of miRNAs that are predicted to target each gene

Based on these research findings, as a demonstration of our hypothesis, we cross-referenced these miRNAs linked to music appreciation and performance with the genes associated with the chemokine signaling pathway (hsa04062) outlined in the Kyoto Encyclopedia of Genes and Genomes (KEGG) database (Fig. 1). Target genes of each miRNAs were found by using the tool miRTargetLink 2.0 [68].

We found that out of the 47 genes comprising this pathway, 22 of them might potentially be influenced by one or more of the miRNAs whose expression is either upregulated or downregulated following exposure to music. Overall, it appears that these miRNAs might exert significant control over the regulation of this pathway, which holds a central position in the regulation of inflammation.

While the positive impacts of visual art on overall well-being have been widely recognized, the exploration of epigenetic effects resulting from exposure to artistic expressions remains an uncharted, although promising, territory.

Indeed, by examining, for example, a pivotal change often linked to engagement with art—the reduction in cortisol blood levels [84]—a foundation for the plausibility of an epigenetic framework can be easily established. The intricate regulation of cortisol and its corresponding receptor, the glucocorticoid receptor, is significantly influenced by epigenetic mechanisms, in particular by DNA methylation [85]. Stress has been correlated with a spectrum of epigenetic alterations [86], which can range from dynamically shifting patterns to enduring imprints on the epigenome. Moreover, the level of cortisol, in turn, modulates the methylome [87], programming stress reactivity in the human brain.

Finally, it is captivating to report the close interplay between exposure to art, neural plasticity, and epigenetic modifications. In this intriguing intersection, the malleability of neural circuits might find resonance with the artistic experiences, while the complex epigenetic changes might orchestrate a symphony of transformation within the brain to shape our well-being. Neural plasticity enables neurons to reshape their architecture in response to both internal and external stimuli, through DNA methylation/demethylation and histone acetylation/deacetylation, which have been extensively reviewed by Nayak *et al*. [88].

## Is happiness signature in the chromatin?

If it is well known that chemical modifications of histones orchestrate a wide array of DNA-guided processes, such as gene transcription, a distinctive histone post-translational modification was recently identified: the serotonylation (i.e. covalent binding of serotonin) of glutamine at position 5 (Q5ser) on histone H3 [89]. This modification has been shown to arrange the dynamics of permissive gene expression. Serotonin, a monoamine neurotransmitter, exerts a well-known and extensive influence over mood regulation, cognitive processes, reward pathways, learning, memory consolidation, and an array of vital physiological functions. Serotonin has been often referred to as the “feel-good” neurotransmitter, is a key player in the intricate web of neurochemical interactions that influence our emotions, and can be modulated by positive experiences, such as music and art [90, 91]. The potential implications of serotonin’s stable binding to DNA, resulting in significant impacts on gene expression, could potentially pave the way for a deeper comprehension of the profound influence that music and art have on our emotional state, leading us closer to unraveling the enigmatic connection between creative stimuli and feelings of happiness.

## Boundaries and prospects of the field: exploring scope and future avenues

The exploration of epigenetics in the context of positive experiences, such as art, music, and beauty, opens up fascinating research that holds promise for deepening our understanding of human well-being and the biological basis of emotions. As we explore the interplay between positive emotions and epigenetic modifications, it becomes evident that this field is still in its early stages. While there are small but intriguing studies that start to suggest connections between epigenetic changes and positive experiences, much more research is needed to establish clear causal relationships and identify the underlying molecular mechanisms. Understanding how exposure to art, music, and beauty can lead to specific modifications in DNA methylation, histone modifications, or even novel epigenetic marks such as serotonin-bound histones could shed light on the mechanisms through which these positive experiences influence human biological responses.

**Figure 2:**
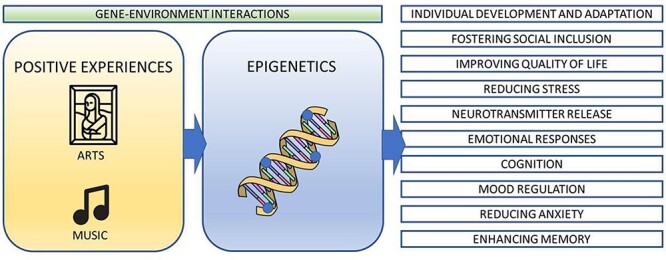
epigenetics as a possible link between individual experiences, epigenetic pattern, and phenotype

The epigenetic pattern serves as a crucial bridge connecting our genes, the unique experiences of each individual, and the resulting phenotype (Fig. 2).

Nevertheless, it is crucial to underline that epigenetic marks tend to exhibit tissue-specific characteristics, reflecting the distinct functional demands and developmental trajectories of each tissue type. While exploring epigenetic traits closely associated with brain function holds great promise, it is essential to acknowledge the potential challenge stemming from this inherent tissue specificity. The complexity of the cell epigenome, with its intricate networks and diverse cell types, underscores the need for caution when extrapolating epigenetic findings from other tissues (e.g. peripheral blood) to the brain.

In further consideration of the complexity of epigenetics, it is important to emphasize the concept of “metastable” effects, wherein lasting molecular changes are often observed only after repeated exposures. While the review has touched upon compelling examples, such as the expression of miRNAs in response to music, it is crucial to acknowledge that these responses may lean more toward the transcriptional spectrum than strictly epigenetic. Therefore, in the future exploration of interventions utilizing art forms, studies should extend beyond immediate molecular responses and embrace a longitudinal perspective. Longitudinal study designs might represent the ideal methodology to discern the enduring memory of both epigenetic and transcriptional alterations associated with outcome measures. Incorporating this temporal dimension into the scientific discussion enriches the impact of art-based interventions on the epigenetic landscape.

Collaborations between researchers from diverse disciplines, including genetics, epigenetics, neuroscience, psychology, and the arts, will be essential to fully unravel the complex interactions between aesthetics, emotions, and epigenetic regulation. Such interdisciplinary efforts can lead to the development of innovative experimental designs, more comprehensive data analysis methods, and a richer interpretation of results.

In our opinion, one open question regards the durability of positive-experiences-induced epigenetic changes. In this light, the field can benefit from more longitudinal studies that track epigenetic changes over time in response to sustained exposure to positive experiences. Examining how regular engagement with the arts, including visual art, music, and many other artistic expressions, can lead to lasting changes in the epigenome and whether these changes are associated with long-term improvements in mental health, well-being, and resilience could provide valuable insights in terms of public health. This aspect becomes even more intriguing when considering the evolving landscape of aesthetic experiences, particularly with the advent of new media and immersive technologies. These novel mediums, by emphasizing emotional engagement more intensely, might potentiate the effects on well-being and potentially induce more profound epigenetic changes. In this light, the field can benefit from more longitudinal studies that track epigenetic changes over time in response to sustained exposure to positive experiences, including those offered by these emerging technologies.

Moreover, if we want to consider visual art and music as potential therapies for the preservation of well-being, an intriguing aspect emerges—one that necessitates contemplation regarding the role of individual education in modulating their efficacy. In fact, it is possible to hypothesize that the attainment of beneficial effects may be contingent upon an individual’s level of sensitivity and depth of education. In this perspective, individuals who possess a higher degree of artistic literacy, having been immersed in the appreciation of various art forms throughout their lives, might be more attuned to the artistic creations, allowing them to extract deeper layers of meaning and emotional resonance and, potentially, amplifying the therapeutic outcomes derived from art and music engagement. Conversely, those with limited childhood exposure or understanding of artistic contexts might not reap the same level of benefits. As we contemplate the potential influence of an individual’s artistic education on the effectiveness of art and music as therapeutic interventions, an additional dimension emerges as this scenario opens the door to a further question: how essential is it to provide education in art and music during childhood?

Childhood has been reported as a critical period for cognitive, emotional, and sensory development. During these formative years, exposure to art and music can shape a child’s perception, creativity, and emotional intelligence. Might art and music also shape the epigenomic landscape, endowing the maturing individual with greater capabilities to navigate their life and ultimately safeguard their future well-being?

“The purpose of art is not the release of a momentary ejection of adrenaline but rather the gradual, lifelong construction of a state of wonder and serenity”

Glenn Gould

## Data Availability

Not applicable.
